# Bioactive Carbon Dots from Clove Residue: Synthesis, Characterization, and Osteogenic Properties

**DOI:** 10.3390/biomedicines13020527

**Published:** 2025-02-19

**Authors:** Hye-Sun Hong, Hee-Jung Park, Ji-Min Lee, Zu-Yu Chen, Tae-Woo Kim, Yong-Seok Seo, Jun-Won Kang, Young-Kwon Seo

**Affiliations:** 1 Department of Biomedical Engineering, Dongguk University, Goyang-si 10326, Gyeonggi-do, Republic of Korea; sugarxxrush@naver.com (H.-S.H.); ireneparkhj@gmail.com (H.-J.P.); kplhnj315@naver.com (J.-M.L.); zuyuchen25@gmail.com (Z.-Y.C.); xodn8876@naver.com (T.-W.K.); 2 Department of Food Science and Biotechnology, Dongguk University, Goyang-si 10326, Gyeonggi-do, Republic of Korea; fmiyl4@naver.com

**Keywords:** carbon dots, clove, green synthesis, bone regeneration, osteogenic differentiation

## Abstract

**Background/Objectives**: Bone regeneration using nanomaterial-based approaches shows promise for treating critical bone defects. However, developing sustainable and cost-effective therapeutic materials remains challenging. This study investigates the osteogenic potential of clove-derived carbon dots (C-CDs) for bone regeneration applications. **Methods**: C-CDs were synthesized using a green hydrothermal method. The osteogenic potential was evaluated in human bone marrow-derived mesenchymal stem cells (hBM-MSCs) and validated using ectopic bone formation and calvarial defect models. **Results**: C-CDs demonstrated uniform morphology (~10 nm) with efficient cellular uptake. In vitro studies showed successful osteogenic differentiation through the upregulation of RUNX2, ALP, COL1A1, and BMP-2 mediated by Wnt/β-catenin/GSK3β and BMP signaling pathways. In vivo models have also demonstrated that C-CDs are effective in promoting bone regeneration. **Conclusions**: These findings establish C-CDs as promising candidates for bone regeneration therapy, offering a sustainable alternative to current treatments. While optimization is needed, their demonstrated osteogenic properties warrant further development for regenerative medicine applications.

## 1. Introduction

Bone regeneration is still a major challenge in clinical medicine, with millions of surgeries performed globally to correct bone abnormalities caused by trauma, disease, or congenital disorders [1]. Despite improvements in tissue engineering, existing treatment techniques have significant limitations. Traditional therapies, such as autografts and allografts, are restricted by donor site morbidity, availability, and the possibility of immunological rejection [2,3]. While recombinant bone morphogenetic protein-2 (BMP-2) has demonstrated encouraging outcomes in bone regeneration, concerns about its high cost, supraphysiological dosage needs, and potential side effects have led to the quest for alternate treatments [4,5]. Recent advancements in nanotechnology have created new opportunities for generating novel therapeutic materials for tissue regeneration [6,7]. Carbon dots (CDs) are a wide family of carbon-based fluorescent nanomaterials that may be classified into various subclasses depending on their structure, content, and production techniques [8]. These subclasses include graphene quantum dots (GQDs), which are characterized by single- or few-layer graphene fragments with lateral dimensions of less than 10 nm, a crystalline structure with sp2 carbon domains, and are typically synthesized through a top–down cutting of graphene sheets or bottom–up molecular assembly [9]. Carbon nanodots (CNDs), also known as carbon quantum dots (CQDs), are quasi-spherical carbon C-CDs with predominantly amorphous carbon cores that are commonly formed by carbonizing organic compounds [10]. Polymer dots (PDs) are a third type, comprised of cross-linked or aggregated polymer chains rich in surface functional groups that are commonly generated via polymerization or polymer aggregation [11].

CDs, for example, have distinct optical and electrical properties as a result of quantum confinement. They exhibit amazing size-dependent photoluminescence and high photostability while providing extra benefits due to their carbon-based composition [12,13]. These nanomaterials have emerged as attractive candidates for biomedical applications due to their distinct physicochemical features, which include small size, excellent biocompatibility, and easily adjustable surface chemistry [14,15]. The development of green synthesis methods for CDs has received a lot of interest, as these approaches provide major benefits in terms of sustainability, cost-effectiveness, and the potential retention of bioactive chemicals from natural precursors [16]. Such ecologically friendly synthesis methods eliminate hazardous chemicals while potentially improving the biological characteristics of the resultant nanomaterials [17,18].

Clove (*Syzygium aromaticum*), a traditional medicinal herb, is well-known for its therapeutic characteristics, which include anti-inflammatory, antioxidant, and antibacterial activities [19]. The high phenolic content and bioactive chemicals in clove, particularly eugenol and flavonoids, make it an appealing precursor for C-CD synthesis [20,21]. These molecules not only serve as carbon sources, but they may also enhance the biological activity of the produced CDs [22].

The goal of this study was to create and test clove-derived CDs as a potential bone regeneration therapy. Our goals were to (1) synthesize and characterize CDs from clove extract; (2) evaluate their cellular uptake; (3) assess their osteogenic potential in vitro using human bone marrow-derived mesenchymal stem cells (hBM-MSCs); and (4) validate their bone regeneration capacity in vivo.

## 2. Materials and Methods

### 2.1. Synthesis of Clove-CDs

**Figure 1:**
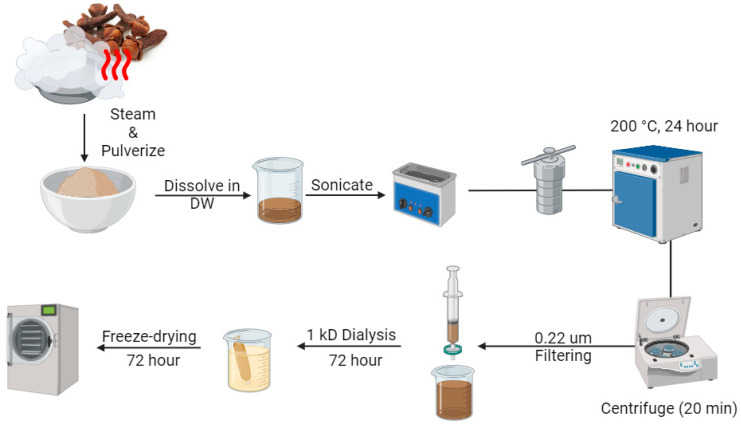
A schematic illustration of the synthesis process for carbon dots (CDs) derived from cloves.

Based on our previous report [23], carbon dots from clove residue were produced using a simple, ecologically friendly, hydrothermal process (Figure 1). Briefly, oil was removed from the clove, ground into powder, and mixed with ultrapure 50% ethyl alcohol (5 g in 50 mL). The mixture was sonicated (24 kHz, 350 W) and hydrothermally treated at 200 °C for 24 h. After cooling and centrifugation (12,000× *g*, 15 min), the supernatant was filtered through a 0.22 μm filter, dialyzed (MW 1000) for 48 h, and freeze-dried to obtain luminous C-CDs powder.

### 2.2. Antioxidant Capacity

The antioxidant capacity of C-CDs was evaluated using an ABTS (2,2′-azino-bis(3-ethylbenzothiazoline-6-sulfonic acid)) radical-scavenging assay and a DPPH radical-scavenging activity.

#### 2.2.1. ABTS Radical-Scavenging Activity

ABTS radical cation (ABTS•+) was produced by reacting 14 mM ABTS with 4.8 mM potassium persulfate and allowing the mixture to stand in the dark at room temperature for 24 h before use. The ABTS•+ solution was diluted with deionized water to obtain an absorbance of 0.70 ± 0.02 at 734 nm. Ascorbic acid (Aa) was used as a positive control. The absorbance was measured at 734 nm using a microplate reader.

#### 2.2.2. DPPH Radical-Scavenging Activity

The DPPH (2,2-diphenyl-1-picrylhydrazyl) radical-scavenging activity was measured using a 0.2 mM DPPH working solution. Samples were mixed with the DPPH solution and incubated for 30 min at room temperature in the dark. As a positive control, 25 uM Aa were used, and the absorbance was measured at 515 nm using a microplate reader.

The radical-scavenging activity for both assays was calculated using the following equation:

Scavenging activity (%) = [(A0 − A1)/A0] × 100

where A0 is the absorbance of the control, and A1 is the absorbance of the sample.

### 2.3. Cell Culture and Treatment

**Figure 2:**
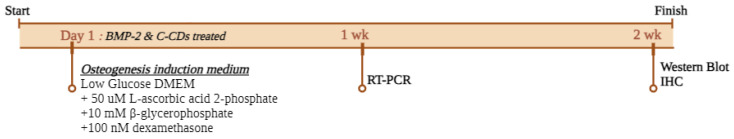
Timeline and culture medium formulation for in vitro evaluation.

Human bone marrow-derived mesenchymal stem cells (hBM-MSCs) were purchased from the American Type Culture Collection (ATCC, Manassas, VA, USA). Human bone marrow-derived mesenchymal stem cells (hBM-MSCs) were grown in conventional growth media containing Dulbecco’s modified Eagle’s medium (DMEM, Welgene, Deagu, Korea) and 10% fetal bovine serum (FBS, Hyclone, Logan, UT, USA). Cells were kept at 37 °C in a humidified environment with 5% CO^2^. To conduct the experiments, the cells were seeded at a density of 1 × 10^5^ cells per well. The treatment groups included growth medium (negative control), osteogenic induction medium (positive control), 20 ng/mL of BMP-2, and 20 µg/mL of C-CDs. To promote osteogenesis, we added 50 µM L-ascorbic acid 2-phosphate, 10 mM β-glycerophosphate, and 100 nM dexamethasone to DMEM. The culture medium was refreshed every 2–3 days for the duration of the experiment. To assess the in vitro osteogenic differentiation capability of C-CDs, we devised a complete experimental approach. The study period was meticulously planned to evaluate the therapeutic efficacy of our therapy technique. Cells were treated as per the experimental conditions 24 h after seeding. We stimulated osteogenic differentiation for one week for qRT-PCR and two weeks for Western blot and IHC analysis (Figure 2).

### 2.4. Quantitative Real-Time PCR

Total RNA was extracted using RNAiso Plus (Takara Bio Inc., Shiga, Japan) according to the manufacturer’s instructions. RNA quantity and quality were measured with a Nanodrop (Nanophotometer P330, München, Germany). Complementary DNA was produced using a reverse transcription master mix, following the manufacturer’s instructions. Quantitative real-time PCR was performed utilizing osteogenic marker-specific primers. The primer sequence pairs are provided in Table 1. β-actin is an internal control. Gene expression was evaluated with the comparative CT (ΔΔCT) technique.

### 2.5. Western Blot Analysis

Protein expression was determined by Western blotting. Cells and tissues were lysed in RIPA buffer enriched with protease inhibitors. The protein content was measured with the BCA test. Equal amounts of protein (20–30 µg) were separated by 10% SDS-PAGE and transferred to PVDF membranes. Membranes were blocked with 5% skim milk and incubated overnight at 4 °C with primary antibodies: anti-β-catenin (sc-7963) in dilution 1:500 (Santa Cruz Biotechnology, Santa Cruz, CA, USA), anti-BMP-2 (BS-1012R) in dilution 1:200 (Bioss Inc., Woburn, MA, USA), anti-GSK-3β (ab131356) in dilution 1:1000 (Abcam, Cambridge, UK), anti-anti-P-p-44/42-MAPK-ERK(1/2) (4370S), and anti-p-44/42-MAPK-ERK. Following secondary antibody incubation, protein bands were detected with the EZ-Westun Lumi Femto (DoGenBio, Seoul, Republic of Korea) and documented with the ChemiDoc XRS+ gel imaging system (Bio-Rad, Hercules, CA, USA). Band intensities were measured using ImageJ (version 1.54, National Institutes of Health, Bethesda, MD, USA).

### 2.6. Histological and Immunohistochemical Analysis

The tissues were collected following euthanasia. Tissues were homogenized using sample buffer (pH 6.8) containing 5% (*v*/*v*) 2-mercaptoethanol and incubated at 95 °C for 5 min. Coverslips were fixed in 4% paraformaldehyde for immunohistochemistry examination and rinsed three times with PBS.

Immunohistochemistry was carried out using specific antibodies against osteogenic markers such as BMP-2 (1:200, Bioss), ALP (1:200, Invitrogen Carlsbad, CA, USA), COL1A1 (1:500, Cell signaling Technology, Danvers, MA, USA), and osteopontin (1:500, Abcam, Cambridge, MA, USA). The DAB (3,3′-diaminodbenzidine) was used for visualization.

### 2.7. Animal Test

The mice were housed under controlled conditions: 12 h light/dark cycle, temperature of 22 ± 2 °C, and relative humidity of 50 ± 10%. The Animal Care and Use Committee of Dongguk University, Seoul, Korea, authorized all experimental methods in this work on 16 October 2024 (IACUC Approval Number: IACUC-2024-022-1). Groups were established based on preliminary dose-finding studies in our laboratory. The doses were selected based on in vitro cytotoxicity studies. Twenty-four 5-week-old male ICR mice (weight 27–29 g) were randomly allocated into three groups, including (1) Control (saline); (2) BMP-2, 25 µg; and (3) C-CDs, 20 mg, for each experiment: (1) the ectopic bone formation model (n = 4) and (2) the calvarial defect model (n = 4).

The mice were anesthetized with Zoletil and Rompun, and a collagen sponge containing BMP-2 or C-CDs was implanted in a subcutaneous pocket for an ectopic bone formation model, while a 4 mm diameter lesion was generated on the right side of the parietal bone under saline irrigation for a calvarial model. After implanting the collagen sponge, the skin was closed using 6–0 monofilament sutures.

### 2.8. Micro-Computed Tomography Measurements

After three weeks of recovery, the mice were euthanized, and tissue blocks containing bone-grafting materials were extracted. After fixation in 10% formalin, the materials were processed and scanned with a micro-CT (Quantum GX2, Revvity, Waltham, MA, USA) running at 90 kV and 80 µA. Following scanning, three-dimensional pictures were reconstructed with Analyze 12.0 (AnalyzeDirect, Overland Park, KS, USA). Bone mineral density (BMD, mg/cc) and bone volume density (BV/TV%) were calculated.

### 2.9. Statistical Analysis

Prism 9 (GraphPad Software, Inc., San Diego, CA, USA) was used for all studies, with the data presented as the mean ± standard deviation (SD). A one-way ANOVA was used to establish statistical significance, which was then followed by Dunnett’s multiple comparisons test. Statistical significance was determined at *p* < 0.05.

## 3. Results

### 3.1. Characterization of C-CDs

**Figure 3:**
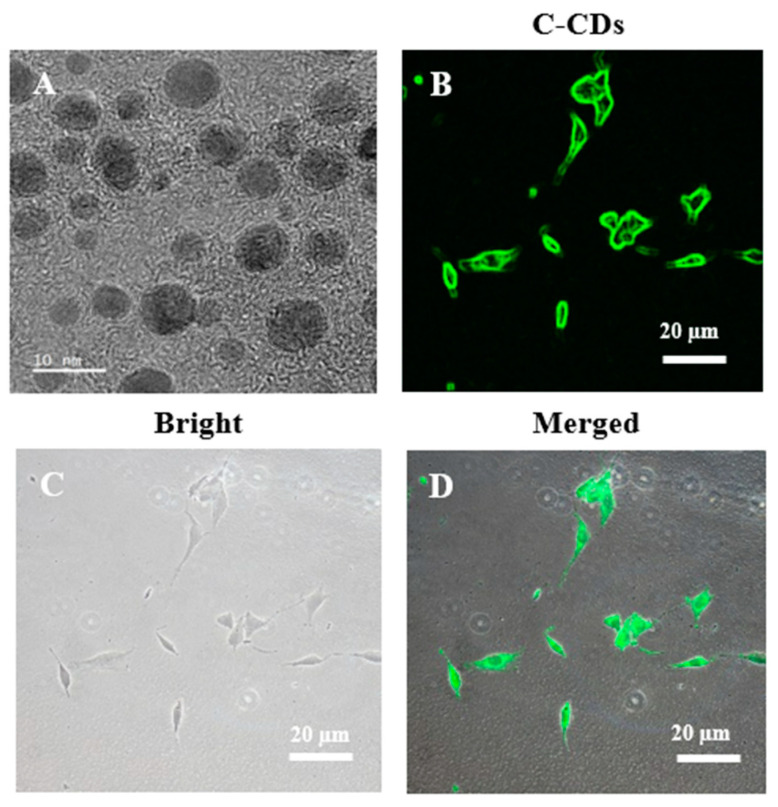
(**A**) High-resolution transmission electron microscopy (HRTEM) micrograph image of clove-derived carbon dots. (**B**–**D**) Endocytosis of C-CDs. Representative images showing endocytosis of C-CDs in cells ((**B**): fluorescence, (**C**): bright field, (**D**): merged images).

The morphological and physicochemical properties of C-CDs were characterized following the same synthesis protocol described in Section 2.1 [23]. The C-CDs exhibited spherical morphology with a uniform size distribution, as confirmed by high-resolution transmission electron microscopy (HRTEM) (Figure 3A). The average particle size and other detailed characterization data of these C-CDs have been extensively documented in the same work. These properties make them suitable for cellular uptake and biological applications, as demonstrated in similar research [23,24].

### 3.2. Cellular Uptake of C-CDs

To explore the cellular uptake of C-CDs, we used their inherent fluorescent characteristics for imaging. Fluorescence microscopy demonstrated that the C-CDs were efficiently internalized by the cells (Figure 3B–D). The fluorescent images (Figure 3B) showed the intracellular accumulation of C-CDs, whereas the bright field images (Figure 3C) highlighted the cellular structure. The merged pictures (Figure 3D) revealed the effective endocytosis of C-CDs, as evidenced by their placement within the cells. The prominent fluorescent signal seen throughout the cytoplasm indicates that C-CDs pierced the cell membrane and were endocytically taken up. This effective cellular internalization is critical for the potential therapeutic application of C-CDs since it ensures that they can reach intracellular targets.

### 3.3. Antioxidant Properties of C-CDs

**Figure 4:**
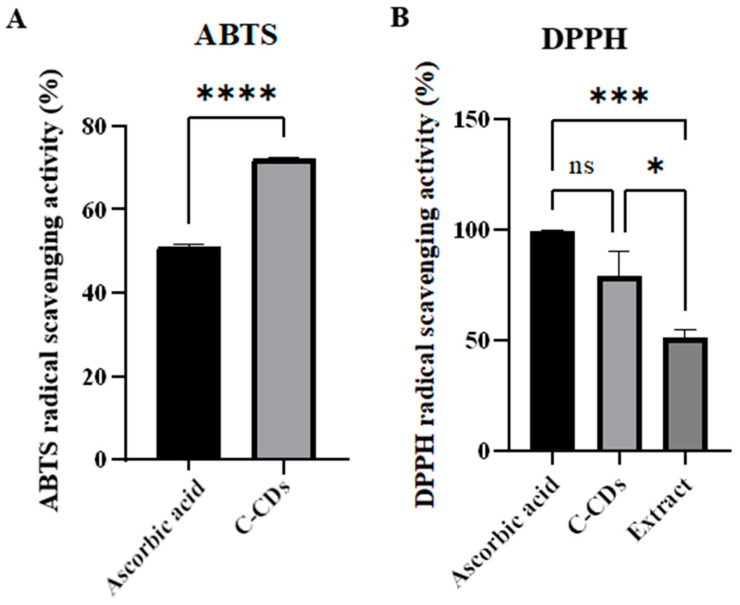
Antioxidant activity of clove-derived carbon dots. (**A**) ABTS and (**B**) DPPH radical-scavenging activity of C-CDs compared with Aa and clove extract. Results are presented as mean ± SD from three independent experiments. * *p* < 0.05; *** *p* < 0.001; **** *p* < 0.0001.

The antioxidant capacity of C-CDs was assessed using both ABTS and DPPH radical-scavenging assays, with Aa serving as a positive control (Figure 4). C-CDs exhibited potent antioxidant activity in both assays, demonstrating concentration-dependent radical-scavenging effects. In the ABTS assay, C-CDs showed significantly higher scavenging activity compared to Aa (72% vs. 50%, *p* < 0.0001). In the DPPH assay, we additionally compared C-CDs with their source material, clove extract, to verify the intrinsic antioxidant properties of the carbon dots. C-CDs demonstrated remarkable DPPH radical scavenging activity of 80%, which was significantly higher than clove extract, which showed 50% scavenging activity (*p* < 0.001). The enhanced antioxidant capacity of C-CDs compared to their source material suggests that the carbonization process successfully improved the antioxidant properties. These results indicate that C-CDs possess strong intrinsic antioxidant properties through multiple mechanisms, potentially contributing to their therapeutic effects in bone regeneration by reducing oxidative stress.

### 3.4. Morphological Changes in Human Bone Marrow-Derived MSCs Following C-CDs Treatment

**Figure 5:**
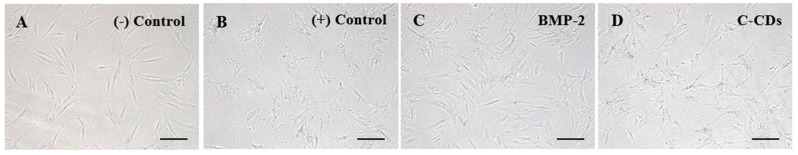
Morphological changes in human bone marrow-derived mesenchymal stem cells (hBM-MSCs) observed at day 10 of osteogenic induction. (**A**) Negative control group showing spindle-shaped fibroblast-like morphology. (**B**) Positive control group with osteogenic induction medium. (**C**) BMP-2 treated group. (**D**) C-CDs treated group. All treatment groups (**B**–**D**) displayed similar fibroblast-like morphology with slight morphological changes compared to the negative control. Scale bar: 100 μm.

Microscopic inspection revealed considerable morphological differences between all experimental groups. On day one, cells in all groups showed the typical fibroblast-like shape of undifferentiated MSCs. On day 10, significant alterations in cellular morphology were detected (Figure 5). Cells in the differentiation medium (Figure 5B–D) exhibited morphological changes similar to those in the growth medium (Figure 5A). However, no significant difference was observed between the positive control group (Figure 5B), the BMP-2-treated group (Figure 5C), and the C-CDs-treated group (Figure 5D).

To comprehensively evaluate the safety profile of C-CDs, we conducted cytotoxicity assessments across various concentrations. The supplementary data (Supplementary Figure S1) presents a thorough analysis of cell viability and potential toxic effects, demonstrating the biocompatibility of our synthesized carbon dots.

### 3.5. Immunohistochemical Analysis for Osteogenesis

**Figure 6:**
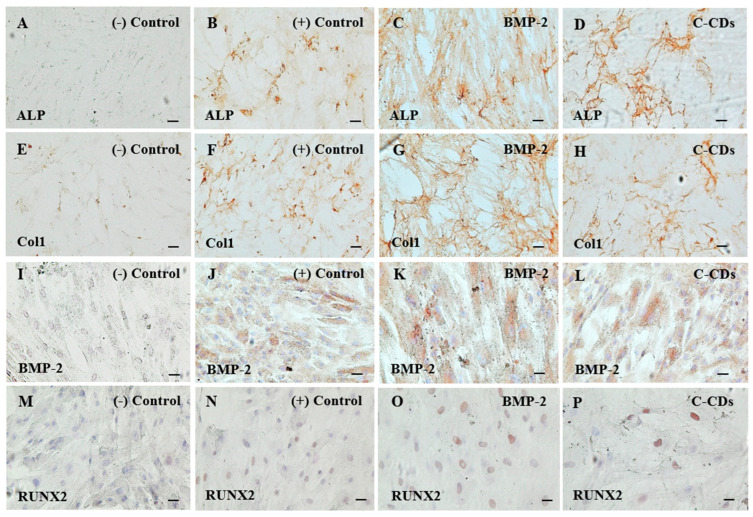
Representative images of IHC staining of the BM-MSCs for osteogenesis markers following the induction of osteogenic differentiation for 14 days. IHC staining was performed to detect the expression of osteogenic markers: ALP (**A**–**D**), Collagen type 1 (**E**–**H**), BMP-2 (**I**–**L**), and RUNX2 (**M**–**P**). Each experimental group is arranged from left to right: negative control, positive control, BMP-2, and C-CDs treatment. Scale bar: 100 μm.

To confirm C-CDs’ osteogenic capacity, we performed an immunohistochemistry (IHC) examination of the key osteogenic markers in hBM-MSCs (Figure 6). Microscopy revealed the geographical distribution and expression levels of four key bone formation markers: ALP, COL1A1 (Coll), bone morphogenetic protein-2 (BMP-2), and RUNX2. Immunohistochemical staining revealed strong expressions of all four markers in both the BMP-2 and C-CDs-treated groups. The expression pattern of ALP, an early indication of osteogenic development, was strongly positive-stained (Figure 6A–D). The high expression of Col1 confirmed active bone matrix deposition (Figure 6E–H). Similarly, the BMP-2 (Figure 6I–L) and RUNX2 (Figure 6M–P) expression levels were significantly higher in both treatment groups. Notably, the staining intensity and distribution patterns in the C-CDs-treated group were equivalent to those in the BMP-2 group, indicating that our synthesized C-CDs have osteoinductive properties.

### 3.6. mRNA Expression Analysis of Osteogenic Differentiation

**Figure 7:**
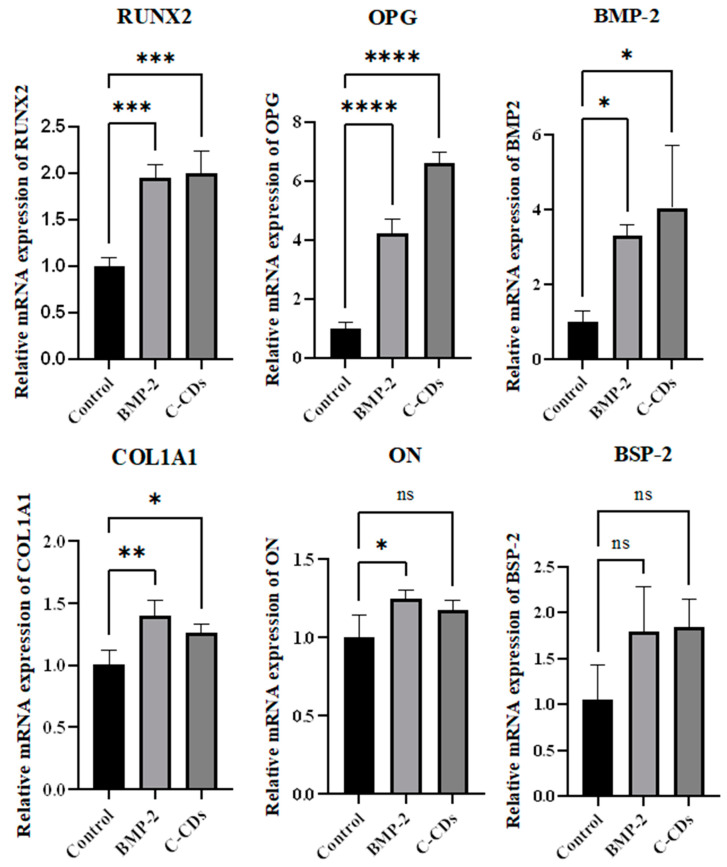
mRNA expression levels of osteogenic markers of BM-MSCs. ns, not significant; * *p* < 0.05; ** *p* < 0.01; *** *p* < 0.001; **** *p* < 0.0001.

To quantify the osteogenic differentiation capacity of C-CDs at the transcriptional level, we used qRT-PCR to examine osteogenic marker gene expression (Figure 7). The gene expression analysis demonstrated that C-CD therapy resulted in considerable elevations of numerous important osteogenic markers. RUNX2 levels increased twofold in both the BMP-2 and C-CD-treated groups compared to the control group. Notably, both treatment groups showed a significant increase in OPG expression, with BMP-2 increasing fourfold and C-CDs increasing more than sixfold. BMP-2 expression was similarly high in both groups, with an approximately 4-fold increase relative to the control group. COL1A1 showed moderate upregulation, with BMP-2 therapy resulting in a 1.5-fold rise and C-CD treatment having a somewhat lower, but still favorable, effect. The expression levels of osteonectin and BSP-2 increased slightly in both treatment groups, but the differences were not statistically significant. These findings show that C-CDs can efficiently enhance osteogenic differentiation by regulating key osteogenic genes, with a considerable effect on OPG and BMP-2 expression.

**Figure 8:**
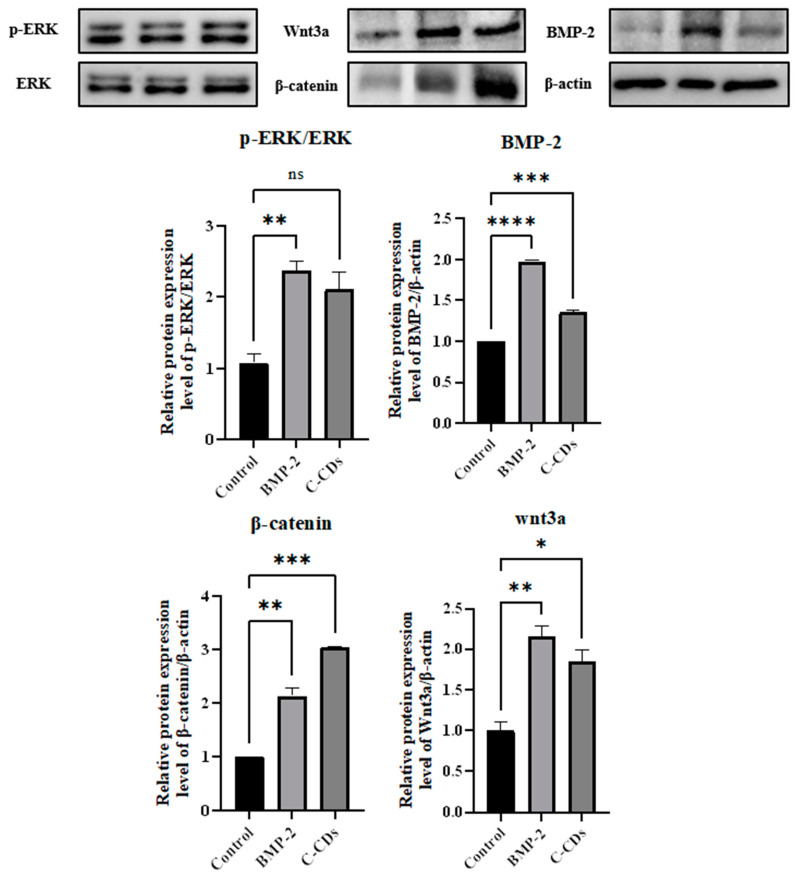
Protein expression levels of osteogenic markers for BM-MSC ns, not significant; * *p* < 0.05; ** *p* < 0.01; *** *p* < 0.001; **** *p* < 0.0001.

In addition, we used a Western blot analysis to confirm the osteogenic differentiation marker in BM-MSCs (Figure 8). The protein expression levels were quantified, and both the BMP-2 and C-CD treatments dramatically increased ERK phosphorylation, with a twofold rise in the p-ERK/ERK ratio compared to the control. BMP-2 therapy increased BMP-2, β-catenin, and Wnt3a protein levels by approximately 2-fold. C-CD administration significantly increased the expression of these proteins, activating the Wnt/β-catenin, ERK, and BMP signaling pathways. These findings indicate that C-CDs promote osteogenic differentiation by activating numerous signaling cascades simultaneously.

### 3.7. Ectopic Bone Formation

**Figure 9:**
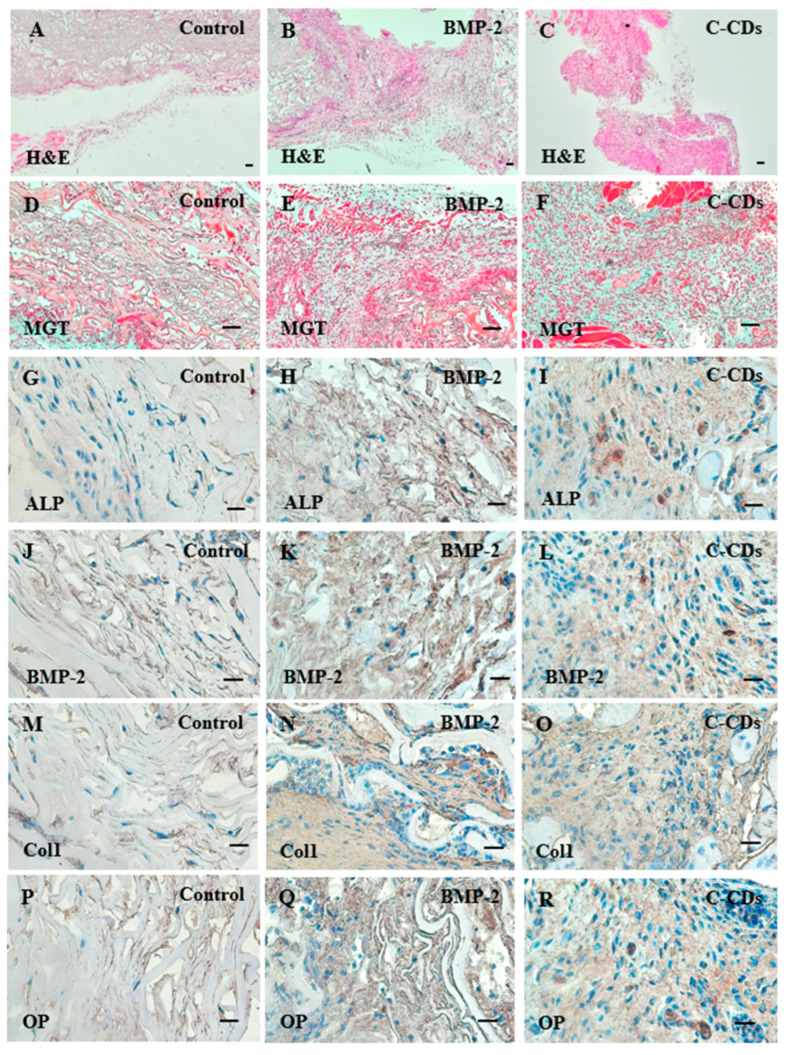
Histological analysis of ectopic bone formation after 2 weeks of subcutaneous implantation. (**A**–**C**) (H and E) staining, (**D**–**F**) Goldner’s trichrome staining, (**G**–**R**) immunohistochemistry (IHC) staining of bone-relative markers. Scale bar: 100 μm.

A histological investigation was undertaken to determine the degree of bone growth within the implanted sponge scaffolds (Figure 9). The specimens were examined using two complementary staining methods: H&E staining to evaluate the general tissue structure and cellular distribution and Masson’s Goldner Trichrome (MGT) staining to precisely visualize bone matrix development and mineralization.

H&E staining indicated varied tissue organization patterns among the treatment groups. While untreated scaffolds showed little tissue infiltration, BMP-2 and C-CD-treated scaffolds showed significant cellular colonization and tissue development (Figure 9A–C). The existence of structured tissue structures in the treated groups showed a successful increase in cell migration. MGT staining supported these findings (Figure 9D–F). Both the BMP-2 and C-CD treatment groups showed collagen matrix deposition, as demonstrated by the high MGT staining, indicating effective osteogenic activity.

To confirm the osteogenic capacity of C-CDs and assess ectopic bone development, we performed an immunohistochemistry (IHC) study of the osteogenic markers in the implanted scaffolds (Figure 9G–R). Microscopy demonstrated the geographical distribution and expression levels of four essential bone formation markers: ALP, BMP-2, Coll, and Osteopontin.

Immunohistochemical staining revealed strong expressions of all four markers in both the BMP-2 and C-CD-treated groups. The expression of ALP, an early hallmark of osteogenic differentiation, was strongly positive throughout the freshly created tissue. Similarly, both treatment groups had significantly higher levels of endogenous BMP-2 expression, showing that osteogenic signaling pathways had been successfully activated. The high expression of Coll demonstrated active bone matrix deposition, while significant osteopontin staining indicated mature bone development. Notably, the staining intensity and distribution patterns in the C-CD-treated group were equivalent to those in the BMP-2 group, indicating that our synthesized C-CDs had osteoinductive properties that promote ectopic bone formation.

### 3.8. Molecular Pathway Analysis of C-CDs-Induced Bone Formation

**Figure 10:**
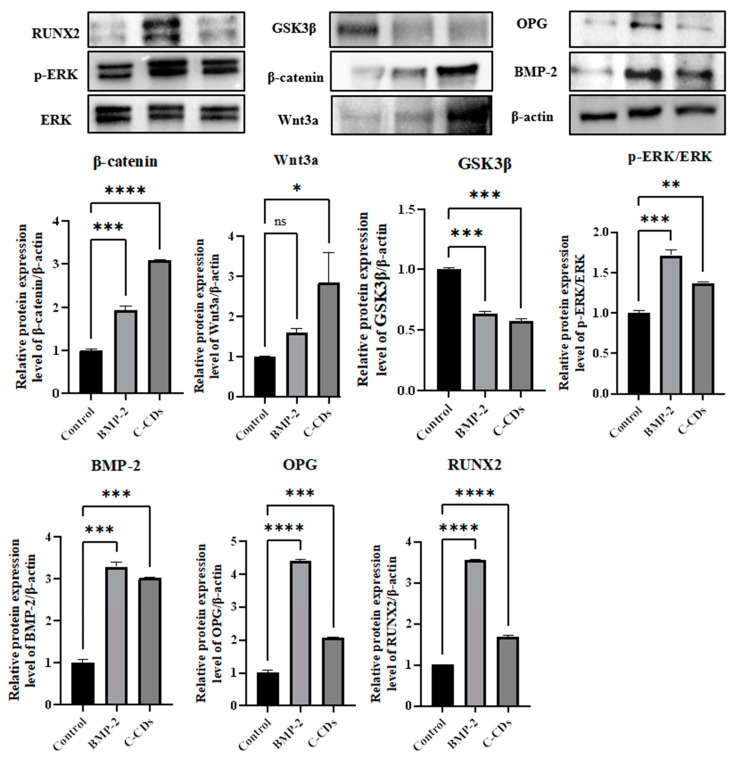
Western blot analysis for osteogenic markers in tissue homogenates obtained from implanted scaffolds. ns, not significant; * *p* < 0.05; ** *p* < 0.01; *** *p* < 0.001; **** *p* < 0.0001.

To validate our findings’ molecular pathways at the tissue level, we performed Western blot analysis on the signaling molecules in the regenerated tissue samples (Figure 10). The findings indicated activation patterns that were both comparable to and dissimilar from those observed in our cellular experiments. The p-ERK/ERK ratio increased somewhat, with BMP-2 treatment causing a more than 1.5-fold rise and C-CD therapy producing a slightly smaller but still significant increase compared to the control. Analysis of the Wnt/β-catenin pathway components revealed considerable upregulation, with β-catenin and Wnt3a exhibiting 1.5- to 2-fold increases in the BMP-2 group. C-CD treatment showed equivalent or even higher levels of activation. BMP-2 expression was also significantly enhanced in both treatment groups, with increases of almost 3-fold relative to the control group.

We found that BMP-2 treatment increased OPG and RUNX2 expression by about fourfold, whereas C-CD treatment increased these markers by twofold. Treatment with BMP-2 and C-CDs reduced GSK3β expression to half that of the control group. Our cellular findings and tissue-level protein expression patterns show that BMP-2 and C-CDs activate the Wnt/β-catenin/GSK3β, ERK, and BMP signaling pathways to promote bone repair.

### 3.9. Quantitative Analysis of Bone Regeneration via Micro-CT

**Figure 11:**
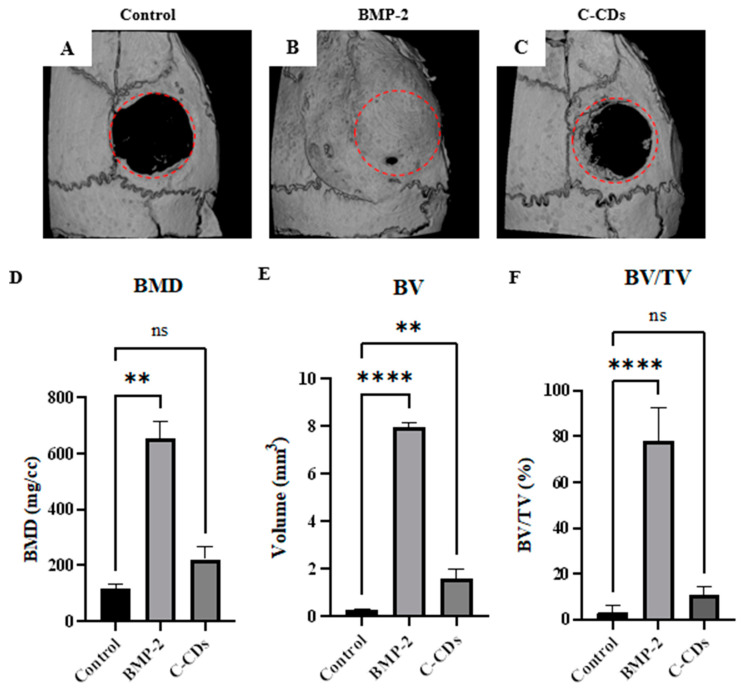
Micro-CT evaluation images (**A**–**C**) and quantitative results of calvarial defect healing after 3 weeks ((**D**): bone mineral density, (**E**): bone volume, (**F**): bone volume/total volume). ns, not significant; ** *p*  <  0.01; **** *p* < 0.0001.

The amount of bone regeneration in the calvarial lesion model was quantified using micro-computed tomography (micro-CT) (Figure 11). As expected, BMP-2 therapy significantly increased bone formation compared to the control group, showing its strong osteogenic capabilities. The C-CD-treated group had marginally higher bone formation than the control group, indicating possible osteogenic capabilities. However, the impact was far less pronounced than that seen with BMP-2 therapy. A quantitative study of bone volume fraction (BV/TV) and bone mineral density (BMD) reveals that the BMP-2-treated group rose more than 6-fold in both data sets, while C-CDs increased around 1.5-fold but not significantly over the control group.

### 3.10. Histological Analysis of Regenerated Bone

**Figure 12:**
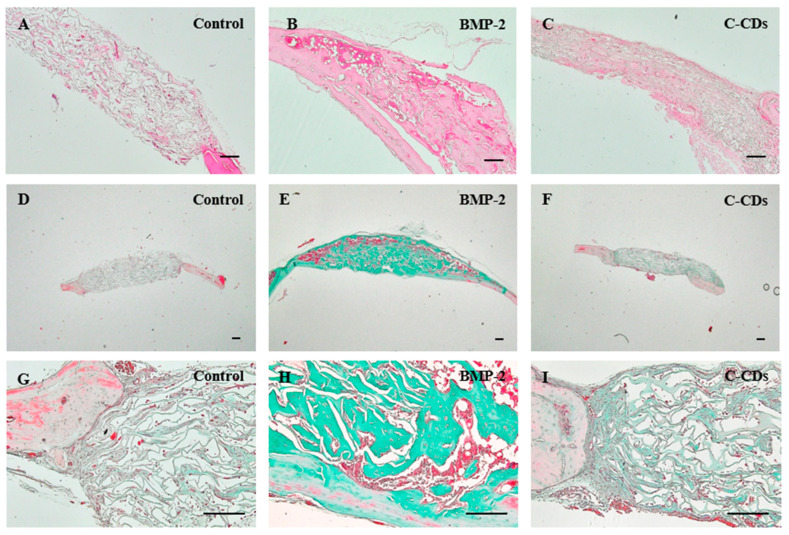
Histological analysis of bone regeneration in calvarial defects. (**A**–**C**) H&E staining of regenerated bone tissue at 3 weeks post-surgery: (**A**) Control, (**B**) BMP-2, and (**C**) C-CDs groups. (**D**–**I**) Goldner’s trichrome staining at 3 weeks post-surgery: (**D**,**G**) Control, (**E**,**H**) BMP-2, and (**F**,**I**) C-CDs groups. Scale bar = 100 μm.

To further evaluate the quality and extent of bone regeneration, we performed histological analysis using H&E and Goldner’s trichrome staining (Figure 12). H&E staining revealed distinct patterns of bone formation across treatment groups. The control group showed minimal bone regeneration, with the defect area primarily filled with fibrous connective tissue. In contrast, the BMP-2 group demonstrated robust bone formation, characterized by excessive tissue growth extending beyond the original calvarial plane, with an average thickness of 1.0 ± 0.1 mm. The C-CD group was able to observe that the ECM was newly secreted compared to the control group, and more ECM formation was confirmed in the transplanted scaffold.

Goldner’s trichrome staining confirmed these observations, with mineralized bone tissue appearing in green. The control group showed minimal mineralization (Figure 12D), while the BMP-2 group displayed extensive but irregular bone formation (Figure 12E). Notably, the C-CDs group demonstrated uniform bone regeneration, with a thickness and structure similar to the surrounding native bone tissue (Figure 12F). These histological findings, combined with our micro-CT analysis, suggest that, while BMP-2 induces more robust bone formation, C-CDs increased bone regeneration compared to the control group.

## 4. Discussion

This study investigated the potential of clove-derived carbon dots (C-CDs) as innovative therapeutics for bone healing. Bone regeneration continues to pose a significant challenge in clinical practice, as existing therapeutic methods are limited by availability, expense, and possible side effects [25,26]. The development of new biomaterials that facilitate bone growth while ensuring safety and cost-effectiveness is a significant area of research [27,28].

The physicochemical assessment of our synthesized C-CDs demonstrated several advantageous characteristics for biological applications. The reproducibility of our green synthesis technique is demonstrated by the consistent spherical morphology and exact size distribution (~10 nm) observed in HRTEM images. This size range is particularly significant, as C-CDs measuring between 5 and 20 nm have demonstrated superior cellular uptake and dissemination [29].

The TEM image revealed that CQDs are uniformly distributed without aggregation and showed a spherical shape with lattice spacing. And XPS analysis revealed that CQDs exhibited C1, N1, and O1 peaks, and the structures were C=C (284.56 eV), C-O (285.79 eV), C=O (287.68 eV), and COOH (288.83 eV). Also, the FTIR spectra of CQDs showed characteristic peaks attributed to C-H (2900 cm^−1^), C=C/C-O (1550 cm^−1^), C=O (1710 cm^−1^), and O-H/N-H (1030 cm^−1^) [23].

These qualities align with previous discoveries that demonstrate uniform size distribution significantly impacts the biological efficacy of C-CDs [30,31], including the strength of the bone microstructure [32,33]. Fluorescence microscopy revealed that C-CDs were effectively absorbed. The detected cytoplasmic localization suggests effective cellular penetration, along with previous studies that demonstrate the significant impact of nanoparticle size and surface properties on cellular uptake [23,34,35].

However, scaling this synthesis process for industrial or clinical applications presents several critical challenges. Industrial-scale production requires severe standardization of raw material sources, precise control of environmental conditions, and development of robust purification methodologies. Variations in clove extract composition, influenced by factors such as geographical origin, harvesting season, and processing techniques, could potentially introduce significant variability in C-CDs’ physicochemical properties. Moreover, achieving high-purity separation techniques that maintain the nanomaterials’ structural integrity and biological functionality remains a substantial technical hurdle.

Future research must focus on developing scalable manufacturing protocols that ensure batch-to-batch consistency, optimize extraction and purification processes, and establish comprehensive quality control metrics. Potential strategies include implementing advanced spectroscopic and chromatographic techniques for precise characterization, developing standardized clove-sourcing protocols, and creating automated synthesis platforms that can maintain reproducibility across different production scales. These technological advancements will be crucial in transitioning C-CDs from promising laboratory-scale research to viable therapeutic and industrial applications.

The morphological changes observed in hBM-MSCs may indicate our C-CDs’ osteogenic potential. The change in morphology is a well-documented marker of osteogenic differentiation [36]. Microscopic observations revealed that the differentiation medium caused morphological alterations in cells when compared to the growth medium, but no variations were seen in response to C-CDs or BMP-2. This is assumed to be due to the short cell culture period, which prevented further confirmation of morphological alterations.

Immunohistochemical analysis confirmed the presence of osteogenic markers, establishing molecular evidence for C-CD-induced osteogenesis. Experimental indicators for measuring osteogenic differentiation were chosen to represent various stages of bone development [37]. ALP, an early measure of osteogenic commitment [38,39], was chosen to evaluate initial differentiation. RUNX2, the main transcription factor of osteogenesis [40,41], sheds light on the molecular programming of osteoblast development. COL1A1 and osteopontin were selected as intermediate and late markers, respectively [42,43,44], to corroborate the process of osteogenic differentiation. The constant elevation of all of these markers in C-CD-treated cells, which is comparable to BMP-2 treatment, significantly confirms our synthetic C-CDs’ osteogenic potential.

For example, Zhu et al. found considerable upregulation of ALP and RUNX2 in Zn2+-doped carbon dots (Zn-CDs) produced using a one-step hydrothermal technique [45]. Shao et al. created citric acid-based carbon dots and ethyl-enediamine using a bottom-up carbonization approach, which enhanced matrix mineralization and had an effect on osteogenic differentiation via the expression of the osteoblast gene markers in BMSCs [46]. Compared to earlier research, we were able to confirm that plant-derived CDs are also beneficial in osteogenic differentiation.

Furthermore, the molecular investigation using qRT-PCR allowed for a quantitative validation of osteogenic marker expression. The increase of RUNX2, BSP-2, and OPG in C-CD-treated cells is especially noteworthy because these markers suggest osteogenic differentiation. Notably, multiple studies have found similar patterns of gene expression in carbon dot-mediated bone repair. Lu et al. developed an injectable hydrogel by conjugating carbon dot nanoparticles onto collagen and cross-linking with genipin via subcutaneous implantation in a nude mouse model [47], resulting in equivalent increases in osteogenic marker expression. Gogoi et al. have confirmed the capabilities of tannic acid-based water-dispersible hyperbranched polyurethane with bio-nanohybrids and carbon dots in bone repair, angiogenesis induction, and mineralization improvement [48].

Our analysis of protein expression and molecular pathways showed intricate signaling networks involved in C-CD-mediated bone healing. The observed alterations in numerous protein expression levels indicate that C-CDs enhance osteogenesis via multiple signaling pathways [49]. Notably, we found concurrent activation of both Wnt/β-catenin, ERK, and BMP signaling pathways, which are critical for osteogenic differentiation. The interplay between these pathways is very important, since recent research has shown that coordinated activation can result in synergistic effects in bone production [50].

The Wnt and BMP signaling pathways are important in bone growth and homeostasis, as they regulate osteoblast differentiation and bone production. C-CDs may stimulate osteogenesis via numerous complementary pathways, as seen by increased β-catenin expression and BMP-2 levels.

Also, our experiment showed considerable downregulation of GSK3β. GSK3β has two roles in bone regeneration. It inhibits Wnt/β-catenin signaling and modulates inflammatory responses [51,52,53]. Controlled inflammation is critical in bone repair because it initiates the restorative process. Recent research has demonstrated that temporary inflammatory responses can enhance bone regeneration [54]. Therefore, C-CDs can improve bone healing by inhibiting GSK3β production via the Wnt/β-catenin signaling pathway.

Furthermore, our findings of enhanced ERK phosphorylation add another layer of molecular activation. The ERK pathway has been shown to play an important role in osteoblast differentiation and bone homeostasis [55,56].

The activation of multiple signaling pathways, including Wnt/β-catenin, ERK, and BMP, reveals a complex molecular mechanism underlying C-CD-mediated osteogenesis. While these pathways are critical for bone formation, their precise interactions and potential off-target effects necessitate comprehensive investigation. The intricate crosstalk between Wnt/β-catenin, BMP, and ERK signaling pathways suggests a multifaceted regulatory network that orchestrates cellular differentiation and bone homeostasis.

The Wnt/β-catenin pathway fundamentally regulates osteoblast differentiation, with its interaction with BMP signaling modulating critical osteogenic gene expression. Simultaneously, the ERK pathway contributes to this process by enhancing transcription factor activation and facilitating matrix mineralization. However, the potential for pathway dysregulation underscores the necessity for meticulous molecular mapping and comprehensive analysis of signaling cascade interactions.

Future research must prioritize elucidating the nuanced molecular mechanisms that govern these pathway interactions, with particular emphasis on identifying potential feedback loops and regulatory checkpoints. Understanding these complex signaling networks is paramount for developing targeted therapeutic strategies that optimize the bone regeneration potential while mitigating unintended cellular responses. The sophisticated interplay between these signaling pathways represents a critical frontier in regenerative medicine research, offering profound insights into the molecular basis of bone tissue engineering.

In these in vivo characteristics, the ectopic bone formation model validated our C-CDs’ osteogenic capability. Previous investigations have shown that various carbon-based materials can successfully create bone in similar models [48,57]. The expression of various bone formation markers in our implanted scaffolds supports effective bone regeneration, which is consistent with the findings from earlier research using carbon-based nanomaterials.

The calvarial defect model results shed light on the therapeutic potential of our C-CDs. While BMP-2 displayed greater bone production, the small improvements reported with C-CDs indicate inherent osteogenic qualities that could be improved with future refinements. Previous research using carbon quantum dots for cerebral bone repair found similar results [58,59]. Based on these findings, additional research into ideal dose regimes and delivery modalities may improve the therapeutic efficacy of our C-CDs. This optimization method may help them maximize their osteogenic potential and produce results comparable to BMP-2 treatment.

While our research demonstrates the promising osteogenic potential of clove-derived carbon dots (C-CDs), a comprehensive examination of their long-term safety profile remains imperative. The inherent biocompatibility suggested by the plant-based origin of these carbon dots presents a notable advantage over synthetic nanomaterials. Previous studies indicate that plant-derived carbon dots exhibit lower cytotoxicity compared to their chemically synthesized counterparts, yet rigorous toxicological investigations are essential.

The exploration of carbon dots (CDs) derived from natural sources has gained significant momentum in recent biomedical research, with multiple studies demonstrating their promising characteristics and potential applications. Chang et al. revealed the potential of fluorescent carbon nanodots synthesized from fresh ginger juice, which exhibited selective inhibition of human hepatocellular carcinoma cells with remarkably low toxicity to normal cells [60]. Selvan et al. further supported this trend by synthesizing carbon quantum dots from Plectranthus amboinicus leaves, showcasing their exceptional photo stability, biocompatibility, and low cytotoxicity [61].

Similarly, Xue et al. demonstrated the versatility of plant-based carbon dots by utilizing Salix wood powder to create nanoparticles with unique light-capturing properties, further emphasizing their environmental adaptability [62]. Yun et al. expanded this narrative by developing carbon dots from Nigella sativa seeds, which exhibited remarkable sensing capabilities and minimal biological interference [63]. Collectively, these studies provide robust evidence supporting the safety, biocompatibility, and multifunctional nature of plant-derived carbon dots, underscoring their potential as innovative nanomaterials in biomedical applications. The consistent findings across these research efforts suggest that natural source-derived carbon dots represent a promising and relatively safe approach to nanomaterial development, with minimal toxicological concerns and a broad potential for therapeutic and diagnostic applications.

Future research must systematically evaluate cellular and tissue-level toxicity across diverse concentration gradients and exposure durations. Critical areas of investigation include comprehensive metabolic pathway analysis, excretion mechanisms, potential immunological responses, and long-term accumulation characteristics. Quantitative assessments of potential inflammatory reactions, cellular uptake kinetics, and biodistribution will be crucial for establishing a robust safety framework. The preliminary findings suggest that C-CDs derived from clove extract may offer a promising, biocompatible approach to bone regeneration. However, extensive preclinical and potential clinical studies are necessary to conclusively validate their therapeutic potential and safety profile. The interdisciplinary nature of these investigations will require collaborative efforts across the toxicology, materials science, and regenerative medicine domains to comprehensively characterize the biological interactions of these novel nanomaterials.

## 5. Conclusions

This study comprehensively investigated the potential of clove-derived carbon dots (C-CDs) as an innovative therapeutic approach for bone regeneration, addressing a critical challenge in regenerative medicine. Our research demonstrated that C-CDs possess remarkable osteogenic properties. Also, our synthesis approach utilizing clove extract provides a sustainable and cost-effective method of nanomaterial production and introduces a potentially biocompatible alternative to conventional bone regeneration strategies.

Our experimental findings highlight the multifaceted potential of C-CDs in bone tissue engineering. The carbon dots exhibited efficient cellular uptake, uniform morphological characteristics, and the ability to activate critical signaling pathways. These pathways play pivotal roles in osteogenic differentiation, suggesting that C-CDs can effectively modulate the cellular processes that are fundamental to bone formation. The observed upregulation of essential osteogenic markers further validates the therapeutic potential of this nanomaterial.

While our experiments revealed promising results, significant optimization is required to fully realize the therapeutic capabilities of C-CDs. Future research must prioritize comprehensive mechanistic studies that elucidate precise molecular pathway interactions, conduct extensive long-term safety assessments to establish complete biocompatibility, and develop targeted delivery mechanisms to maximize therapeutic efficacy. The interdisciplinary nature of this research bridges nanotechnology, materials science, and regenerative medicine, representing a significant advancement in developing sustainable therapeutic strategies.

By leveraging the inherent properties of plant-derived nanomaterials, we have opened new avenues for developing cost-effective, biocompatible therapeutic solutions. The integration of green synthesis techniques, sophisticated molecular understanding, and innovative nanomaterial design offers an exciting pathway toward more effective and accessible regenerative medicine strategies. Although challenges remain in translating these laboratory findings into clinical applications, our research provides a robust scientific foundation for future investigations into bone regeneration and nanomaterial-based therapeutic approaches.

## Figures and Tables

**Table 1:** Sequence of primers used in this study.

Gene	Primer	Sequence 5′―3′
*GAPDH*	Forward	GAGTCAACGGATTTGGTCGTATTG
	Reverse	GCTGTAGCCAAATTCGTTGTC
*RUNX2*	Forward	CCACCGAGACCAACAGAGTC
	Reverse	GTCACTGTGCTGAAGAGGCT
*BMP-2*	Forward	TCGAAATTCCCCGTGACCAG
	Reverse	GAATCCATGGTTGGCGTGTC
*BSP-2*	Forward	AAGGGCACCTCGAAGACAAC
	Reverse	CCCTCGTATTCAACGGTGGT
*OPG*	Forward	CGCCTCCAAGCCCCTGAGGT
	Reverse	CAAGGGGCGCACACGGTCTT
*COL1A1*	Forward	GTTTGGATGGTGCCAAGGGA
	Reverse	CAGTAGCACCATCATTTCCACG
*ON*	Forward	ATTGACGGGTACCTCTCCCA
	Reverse	TCCAGGTCACAGGTCTCGAA

## Data Availability

The data supporting this study’s findings are available from the corresponding author upon reasonable request.

## Supplementary Materials

The following supporting information can be downloaded at: https://www.mdpi.com/article/10.3390/biomedicines13020527/s1, Figure S1: cell viability assessment of C-CDs in different cell types.
