# Supplementary figures and images for: Bioactive Carbon Dots from Clove Residue: Synthesis, Characterization, and Osteogenic Properties
Source: Biomedicines. 2025 Feb 19;13(2):527. doi: 10.3390/biomedicines13020527 (PMC11852471; doi:10.3390/biomedicines13020527)

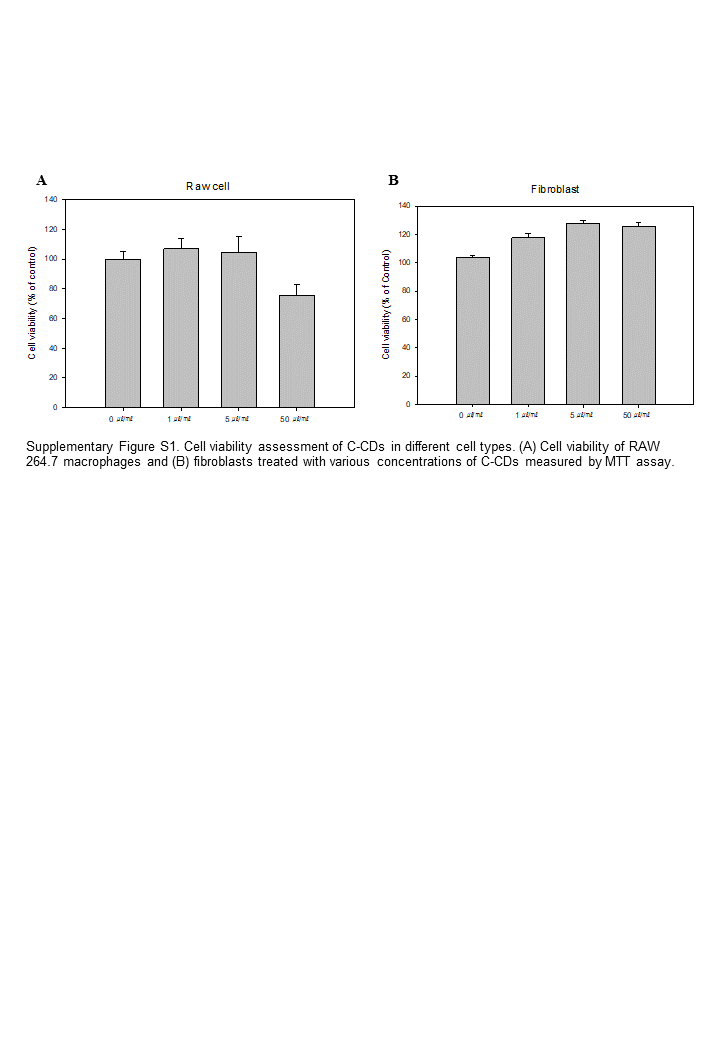

Supplement: Supplementary file 1 [file biomedicines-13-00527-s001.zip › biomedicines-3455479-supplementary.png]
